# Severe postpartum sepsis with prolonged myocardial dysfunction: a case report

**DOI:** 10.1186/1752-1947-4-318

**Published:** 2010-10-08

**Authors:** Michael A Mazzeffi, Katherine T Chen

**Affiliations:** 1 Department of Anesthesiology Mount Sinai School of Medicine One Gustave L Levy Place Box 1010 NY, NY 10029 USA; 2 Department of Obstetrics and Gynecology Mount Sinai School of Medicine 1176 5th Ave E Level NY, NY 10029 USA

## Abstract

**Introduction:**

Severe sepsis during pregnancy or in the postpartum period is a rare clinical event. In non obstetric surviving patients, the cardiovascular changes seen in sepsis and septic shock are fully reversible five to ten days after their onset. We report a case of septic myocardial dysfunction lasting longer than ten days. To the best of our knowledge, this is the first report of prolonged septic myocardial dysfunction in a parturient.

**Case presentation:**

A 24 year old Hispanic woman with no previous medical history developed pyelonephritis and severe sepsis with prolonged myocardial dysfunction after a normal spontaneous vaginal delivery.

**Conclusions:**

Septic myocardial dysfunction may be prolonged in parturients requiring longer term follow up and pharmacologic treatment.

## Introduction

Septic shock in obstetric patients is a rare clinical event. The estimated incidence is one in 8,338 deliveries [1]. In one case series of 18 obstetric patients that developed septic shock, two thirds of patients were in the antepartum period and one third, postpartum. The most common cause of septic shock was pyelonephritis, and the most common pathogen isolated was *Escherichia coli*. Myocardial dysfunction was common among these patients. Another series included ten obstetric patients with septic shock and five patients (50%) were found to have evidence of left ventricular dysfunction [2]. All patients had improvement in ventricular function during their hospitalization. Neither of these series, however, provided information on the duration of myocardial dysfunction in obstetric patients. In surviving non obstetric patients, myocardial dysfunction has been shown to be fully reversible in five to ten days after its onset [3]. We present a case of a parturient with prolonged septic myocardial dysfunction leading to symptomatic heart failure.

## Case presentation

A 24 year old nulliparous Hispanic woman with no past medical history presented at 40 weeks of gestation in active labor. Her antenatal course had been uncomplicated and her labor was uneventful.

12 hours after delivery, she complained of chills, diaphoresis, and right sided back pain. She had a fever of 40°C, a heart rate of 110 beats per minute, a blood pressure of 136/85mmHg, and a respiratory rate of 20 breaths per minute. On examination, she had marked right costovertebral tenderness. Laboratory tests showed a white blood cell count of 18,000 white blood cells/μL and urinalysis of 3+ blood, 3+ leukocyte esterase, and 49 white blood cells per high powered field. Our patient was started on intravenous antibiotics for presumed pyelonephritis.

36 hours after delivery, she complained of extreme difficulty breathing, non productive cough, and generalized malaise. Her fever had risen to 40.5°C. Her heart rate was 136 beats per minute, blood pressure of 136/82mmHg, respiratory rate of 32 breaths per minute and arterial oxygen saturation 76% on room air. A chest film showed poor aeration in both lung bases and large bilateral pleural effusions. Blood cultures and urine cultures both grew *E. coli*.

40 hours after delivery, she was intubated. A 12 lead electrocardiogram showed sinus tachycardia with no ischemic changes. Troponins levels were not elevated and a transthoracic echocardiogram showed a depressed ventricular ejection fraction of 35%. Both the right and left ventricle appeared hypokinetic with normal end diastolic diameters. Valvular function was normal. Chest computed tomography (CT) with intravenous contrast showed significant bilateral airway disease and no evidence of a pulmonary embolus.

On postpartum day five, our patient was extubated. On the eighth postpartum day, a cardiologist evaluated her for continued complaints of shortness of breath, orthopnea, and poor exercise tolerance. Metoprolol and furosemide were started for systolic heart failure; however, she continued to have persistent dyspnea and poor exercise tolerance despite pharmacologic treatment. Ten days postpartum she had a second transthoracic echocardiogram which showed a persistent decreased ejection fraction of 35%. 11 days postpartum, she was discharged home on oral antibiotics, furosemide, and metoprolol.

On postpartum day 21, our patient presented to the Emergency Department with complaints of heavy vaginal bleeding for which she received treatment with intravenous crystalloid solution. During this visit, she reported adherence with her medications, but continued dyspnea and poor exercise tolerance. She could walk only one block without developing shortness of breath and was having considerable difficulty ascending three flights of stairs. Her vital signs at that time were temperature of 36°C, respiratory rate of 16 breaths per minute, heart rate of 57 beats per minute, and blood pressure of 120/72mmHg. She had a normal blood hematocrit and did not require blood transfusion. She was advised to continue her diuretic and beta blocker and discharged from the emergency department with a plan to follow up as an outpatient. Unfortunately, she did not return to our medical center after this visit and we were unable to contact her despite numerous attempts.

## Discussion

Cardiovascular dysfunction is a defining feature of severe sepsis and septic shock [4]. Typically, myocardial dysfunction and decreased ventricular ejection fraction occurs within the first 24 to 48 hours after the onset of sepsis. In surviving non obstetric patients, these changes have been shown to be fully reversible in five to ten days.

The mechanisms of myocardial dysfunction during sepsis are not fully understood, but have been elucidated over the last several decades. One early hypothesis was that coronary blood flow might be compromised during sepsis leading to decreased myocardial performance. However, it has been shown that coronary flow actually increases during sepsis. Another hypothesis was that circulating myocardial depressant substances are responsible for myocardial dysfunction during sepsis. This hypothesis has been supported by evidence showing serum from patients with sepsis can depress *in vitro *contraction of animal muscle fibers [5]. Two of the circulating myocardial depressant substances are thought to be the cytokines, tumor necrosis factor and interleukin 1B [6]. These molecules can cause myocardial depression through a mechanism involving high levels of intracellular cyclic guanosine monophosphate (GMP) and nitric oxide [7]. Other mechanisms that have been implicated in septic myocardial dysfunction include: apoptosis, mitochondrial dysfunction, and abnormal intracellular calcium homeostasis in cardiac myocytes. Increased matrix metalloproteinase activity and autonomic nervous system dysfunction have also been described as possible etiologies [8]. During pregnancy there are normal physiologic changes in the human cardiovascular system. These include an increase in the blood volume, an increase in heart rate, a decrease in systemic vascular resistance, and an increase in cardiac output. Left ventricular function is normal during pregnancy and ejection fraction is not significantly decreased. In this case, myocardial dysfunction from sepsis was prolonged in our obstetric patient leading to systolic heart failure. The mechanism for this prolonged myocardial dysfunction is not clear. However, previous animal studies have shown that during the third trimester of pregnancy there is an enhanced non specific immunological reaction to endotoxin which leads to higher levels of circulating cytokines such as tumor necrosis factor alpha and interleukin-1. Both molecules are myocardial depressant substances and could be partly responsible for the prolongation of septic myocardial dysfunction in parturients [9]. Other cellular mechanisms of septic myocardial dysfunction may also be potentiated by the enhanced immunological response to sepsis during pregnancy, but this is speculative.

The differential diagnosis of heart failure in a parturient includes peripartum cardiomyopathy, myocarditis, and other cardiomyopathies such as viral, familial, dilated, hypertrophic, or drug related. In this case, our patient did not meet the consensus definition for peripartum cardiomyopathy which requires that there be no identifiable cause for cardiac failure other than pregnancy [10]. She also did not have a dilated left ventricle, which is commonly found in peripartum cardiomyopathy [11]. In another previously reported case, a parturient developed septic myocardial dysfunction leading to heart failure, which mimicked a peripartum cardiomyopathy [12]. In this case, the patient developed severe sepsis from endometritis. Myocardial dysfunction was profound with a decrease in ejection fraction to 28%. The patient had symptoms of shortness of breath and lower extremity edema. However, by postoperative day number 11, myocardial function had entirely returned to normal and there were no long term sequelae.

It also seemed unlikely that our patient had pre existing cardiac disease which was exacerbated by severe sepsis because she reported previous good health before and during her pregnancy. We did not believe myocarditis was likely because of the normal troponin level and electrocardiogram. An endomyocardial biopsy could have been performed, but the rate of a positive biopsy is low even in those with high suspicion for myocarditis and the procedure is invasive [13].

The treatment of septic myocardial dysfunction involves aggressive intravenous pre load resuscitation with either crystalloid or colloid, the use of ionotropic and vasopressor drugs for treating arterial hypotension, and possibly the use of simvastatin which inhibits 3-hydroxy-methylglutaryl coenzyme A pathways [14]. Interestingly, our patient was not hypotensive and treatment with ionotropes or vasopressors was not required. She did initially require crystalloid resuscitation in the intensive care unit. Septic myocardial dysfunction in our patient manifested as symptoms of congestive heart failure and dyspnea on exertion. For this reason, she was treated with both a beta blocker and a diuretic because these are first line drug therapies for systolic heart failure. Beta blockers typically increase ejection fraction by five to ten percent in patients with systolic heart failure and improve symptoms [15]. Angiotensin converting enzyme inhibitors are also a first line drug therapy for systolic heart failure but were not started in our patient because of their possible teratogenic effects in her developing infant [16].

A limitation of this report is that we did not measure levels of myocardial depressant substances such as tumor necrosis factor and interleukin 1B as measurement is not standard in clinical practice. In addition, there is lack of long term follow up with our patient. However, our patient had confirmed persistent myocardial dysfunction on a transthoracic echocardiogram on postpartum day ten and symptoms of heart failure on postpartum day 21. We believe these facts indicate that septic myocardial dysfunction may be prolonged in parturients.

## Conclusions

This case demonstrates that myocardial dysfunction from severe sepsis may be prolonged in parturients requiring longer term follow up and pharmacological therapy for symptom relief.

## Abbreviations

CT: computed tomography; GMP: guanosine monophosphate.

## Competing interests

The authors declare that they have no competing interests.

## Consent

Written informed consent for publication could not be obtained despite all reasonable attempts. Every effort has been made to preserve the anonymity of the patient and there is no reason to think she would object to publication.

## Authors' contributions

Both authors contributed to the patient's clinical care and manuscript preparation. Both authors read and approved the final manuscript.
